# Tau Filament Self-Assembly and Structure: Tau as a Therapeutic Target

**DOI:** 10.3389/fneur.2020.590754

**Published:** 2020-11-12

**Authors:** Sebastian S. Oakley, Mahmoud B. Maina, Karen E. Marshall, Youssra K. Al-Hilaly, Charlie R. Harrington, Claude M. Wischik, Louise C. Serpell

**Affiliations:** ^1^Sussex Neuroscience, School of Life Sciences, University of Sussex, Brighton, United Kingdom; ^2^College of Medical Sciences, Yobe State University, Damaturu, Nigeria; ^3^Chemistry Department, College of Science, Mustansiriyah University, Baghdad, Iraq; ^4^School of Medicine, Medical Sciences and Nutrition, University of Aberdeen, Aberdeen, United Kingdom; ^5^TauRx Therapeutics Ltd., Aberdeen, United Kingdom

**Keywords:** tau, tauopathies, Alzheimer's disease, tau aggregation inhibitors, *in vitro* models, self-assembly, filaments

## Abstract

Tau plays an important pathological role in a group of neurodegenerative diseases called tauopathies, including Alzheimer's disease, Pick's disease, chronic traumatic encephalopathy and corticobasal degeneration. In each disease, tau self-assembles abnormally to form filaments that deposit in the brain. Tau is a natively unfolded protein that can adopt distinct structures in different pathological disorders. Cryo-electron microscopy has recently provided a series of structures for the core of the filaments purified from brain tissue from patients with different tauopathies and revealed that they share a common core region, while differing in their specific conformation. This structurally resolvable part of the core is contained within a proteolytically stable core region from the repeat domain initially isolated from AD tau filaments. Tau has recently become an important target for therapy. Recent work has suggested that the prevention of tau self-assembly may be effective in slowing the progression of Alzheimer's disease and other tauopathies. Here we review the work that explores the importance of tau filament structures and tau self-assembly mechanisms, as well as examining model systems that permit the exploration of the mode of action of potential inhibitors.

## Introduction

Newly synthesized and unfolded proteins must undergo a carefully controlled folding process to produce their specific biologically active conformation, known as the native state. This is a specific 3D structure required for correct protein function and is encoded in the amino acid sequence (1). This folding process can be thought of as following an energy landscape whereby the unfolded polypeptide folds to form the native state, the most thermodynamically stable configuration for the polypeptide (2). The unfolded proteins at the top of the landscape are initially driven by hydrophobic collapse to form secondary structure and further intramolecular contacts, such as covalent bonds and hydrogen bonds, help the proteins reach their native state (3, 4). Alternatively, proteins can form misfolded intermediates, which can go on to generate a pathological conformation leading to the formation of amyloid fibrils. However, multiple diseases are associated with stable aggregates that have formed as a result of the assembly of misfolded proteins and dysfunctions in the protective mechanisms, such as the molecular chaperones (1, 2, 5).

Contrary to the native state, where hydrophobic residues are protected, misfolded proteins have exposed hydrophobic residues. This facilitates the formation of hydrophobic interactions between misfolded proteins and drives the self-assembly of proteins into protein amyloid (6). Amyloid fibrils are composed of assembled peptides formed of β-sheets with a typical cross-β structure, which is common for all amyloid, regardless of the amino acid sequence and native structure of the precursor protein (7). The conversion of soluble, monomeric protein into amyloid fibrils includes the production of partially folded intermediates, which have become a focus for understanding pathogenesis of disease in recent years (8–11). These include dimers, trimers, tetramers, oligomers, and protofilaments (12–15). Mature amyloid fibrils are made of several individual protofilaments (16). The accumulation of amyloid fibrils, and their related intermediates, are strongly associated with cellular dysfunction and are common pathological hallmarks for neurodegenerative diseases, such as Alzheimer's disease (AD), Parkinson's disease and Huntington's disease.

Each neurodegenerative disease has its own disease-specific protein that forms disease-specific amyloid fibrils. Nevertheless, there is a group of neurodegenerative diseases, known as tauopathies, that are characterized by the presence of amyloid fibrils consisting entirely of self-assembled tau protein. Filaments that accumulate in the various tauopathies differ in the specific fibril structure as a result of tau aggregation, but this highlights an opportunity where an understanding of tau self-assembly in one tauopathy, may benefit our research in investigating another tauopathy.

## TAU Protein in Disease

### Physiological Expression and Function

**Figure 1:**
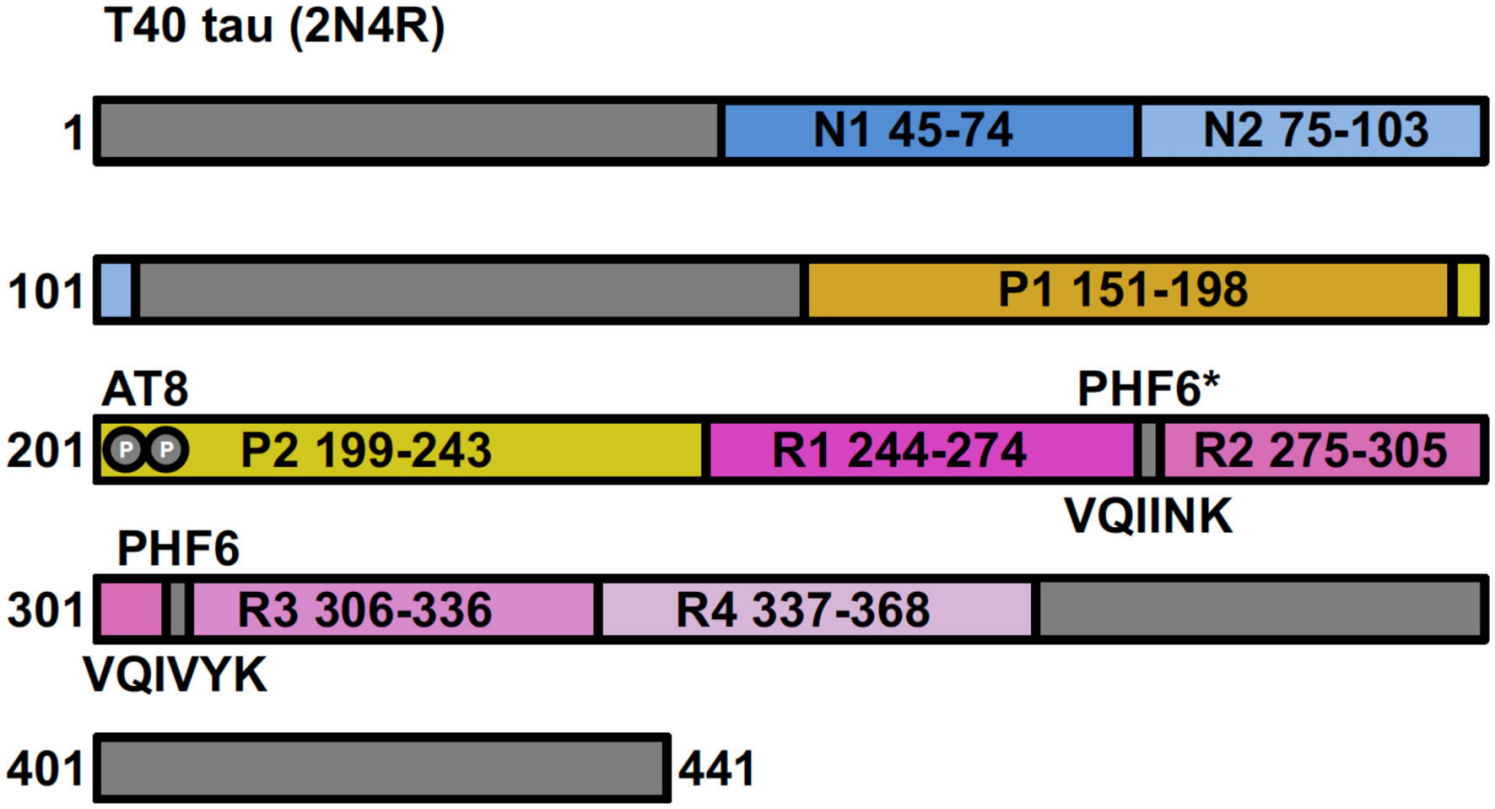
Full-length tau (isoform 2N4R) containing two N-terminal inserts (N1 and N2), two proline-rich regions (P1 and P2) and four microtubule-binding repeats (R1–R4). Antibody AT8 recognizes an epitope dependent on phosphorylation at Ser201 and Thr205. The 6 residue PHF (306–311) and PHF6* (17–22) fragments are from repeat regions R3 and R2, respectively.

Human tau protein is encoded by the *MAPT* gene located on chromosome 17 and is expressed in the central nervous system as a family of six isoforms. These isoforms are the product of alternative mRNA splicing of transcripts from *MAPT*, resulting in proteins of varying amino acid lengths from 352 to 441. They differ by the presence of zero, one or two N-terminal inserts encoded by exons 2 and 3 (0N, 1N, 2N), and the inclusion or absence of the second 31 amino acid microtubule-binding repeat (R2) in the C-terminal half (encoded by exon 10). Inclusion of exon 10 results in the production of three tau isoforms each having four repeats (4R) and the absence of exon 10 produces three isoforms having only three repeats (3R) (Figure 1) (23, 24).

Tau is a family of microtubule-associated proteins responsible for promoting and stabilizing the microtubule network (25, 26). These functions are facilitated by the microtubule-binding domain in the C-terminal domain, with 4R isoforms shown to be better at promoting microtubule assembly and binding to microtubules than the 3R isoforms (27). Microtubules are essential for a range of cellular processes for the maintenance of neuronal morphology, such as intracellular transport of organelles, cell division and signal transduction (28). It is important to note that the different isoforms may be differentially localized depending on the developmental stages, tissues, cell lines, brain regions and intracellular compartments (29–34). For instance, in the murine brain, 1N tau isoforms are overexpressed in the pituitary gland, compared to the cortex or hippocampus and under-expressed in the olfactory bulb. The 2N isoforms are enriched in the cerebellum compared to any other brain region, but are under-expressed in the olfactory bulb; the 0N isoforms are the predominantly expressed isoforms in the olfactory bulb and cortex (34). Intracellularly, in the murine brain, 1N isoforms predominate in the nuclear fraction and 0N isoforms predominate in the cell body/axons (34). In human immortalized cells, like SH-SY5Y neuroblastoma cells, tau may localize to the nucleus or cytoplasm depending on whether the cells are differentiated or not (35). Moreover, it has been shown that the 3R isoform of tau is preferentially localized to the nucleolus (29).

Tau has been observed to bind nucleic acids, suggesting the involvement of tau in chromatin remodeling. This interaction with DNA indicates that tau proteins may play a role in protection against reactive oxygen species (36, 37), ribosome stability and miRNA activity (38). It has also been shown that tau plays a role in nucleolar transcription and participates in the nucleolar stress response (39, 40). These roles seem to be important in the pathogenesis of tauopathies. For example, tau pathology has been shown to impair nucleocytoplasmic transport in AD and frontotemporal dementia (41, 42), induce nuclear envelope invagination in AD (43, 44), induce neurodegeneration through global chromatin relaxation (45), and transposable element dysregulation in AD (46). These studies emphasize the importance of a nuclear role for tau in tauopathies.

### Tauopathies

Tauopathies are clinically, biochemically and morphologically heterogenous neurodegenerative diseases characterized by the deposition of abnormal aggregates of tau in the brain (47). In each tauopathy, tau self-assembles via the repeat domains to form a disease-specific conformation, which associates to form disease-specific amyloid fibrils. It is widely recognized that the accumulated tau pathology seen in tauopathies is directly associated with cognitive decline and clinical dementia in patients suffering with these diseases (48–56). The importance of tau in neurodegeneration was emphasized when it was discovered that dominantly inherited mutations in *MAPT* cause frontotemporal dementia with parkinsonism-linked to chromosome 17 (FTDP-17) (57–59). Unlike AD, FTDP-17 is not characterized by the presence of amyloid β (Aβ) peptide plaques but patients exhibit cognitive decline. Pathologically aggregated tau is therefore sufficient, in the absence of Aβ to result in neurodegeneration and dementia in the absence of Aβ (60).

Tauopathies include AD, frontotemporal dementia (FTD: which includes Pick's disease (PiD) as a sub-type), chronic traumatic encephalopathy (CTE), corticobasal degeneration (CBD), primary age-related tauopathy (PART), progressive supranuclear palsy (PSP), and argyrophilic grain disease (AGD). Table 1 outlines key features of the tau aggregates associated with different tauopathies. They can be classified into primary or secondary depending on whether tau aggregates represent the sole molecular lesion, or if the disease is associated with other pathological features, such as Aβ plaques in AD (73). Tauopathies can be further distinguished by the tau isoforms present in their filaments, with AD and CTE comprising all six isoforms (3R and 4R), CBD, PSP, and AGD only comprising only 4R isoforms, and PiD only containing 3R isoforms (67).

**Table 1:** List of tauopathies and details of their associated tau pathology.

**Tauopathy**	**Primary or secondary tauopathy**	**Tau pathology**	**Filaments**	**Filament core structure (Figure 2)**	**Tau isoforms in filaments**
Alzheimer's Disease (AD) (62, 63)	Secondary (with amyloid-beta plaques)	Neurofibrillary tangles (NFTs)	Paired helical filaments (PHFs): longitudinal spacing between crossovers of 65–85 nm and a width of 7 nm at the narrowest parts and 15 nm at the widest section Straight filaments (SFs): 10 nm wide with crossovers distances ranging from 70–90 nm	Alzheimer fold: Identified by Fitzpatrick and colleagues, consisting of residues 306-378 of 3R and 4R tau (all R3 and R4 repeats, and 10 amino acids after R4). Eightβ-strands adopting a C-shaped architecture	3R/4R
Pick's Disease (PiD)/Frontal temporal dementia (FTD) (64)	Primary	Pick bodies	Narrow Pick filaments (NPFs): helical twist with a crossover distance of ~100 nm and a width of 5–15 nm Wide Pick filaments (WPFs): helical twist with a crossover distance of ~100 nm and a width of 5–30 nm	Pick fold: Identified by Falcon and colleagues, consisting of residues 254–378 of 3R tau (C-terminal part of R1 repeat, all R3 and 4R repeats, and 10 amino acids after 4R). Nine β-strands pack together in a hairpin-like fashion	3R
Chronic traumatic encephalopathy (CTE) (65)	Primary	NFTs and glial tangles (GTs)	CTE type I filaments: Predominant helical filaments widths of 20–25 nm and crossover spacing of 65–80 nm. CTE type II filaments: pronounced helical twists with widths of 15–30 nm. Paired helical filaments (PHFs)	CTE fold: Identified by Falcon and colleagues, consisting of residues 274–379 for 3R tau, 305–379 for 4R tau. Eight β-strands adopting a C-shaped architecture in a more open configuration than the Alzheimer fold, consisting of a hydrophobic cavity	3R/4R
Corticobasal degeneration (CBD) (66)	Primary	NFTs, coiled bodies, argyrophilic threads and astrocytic plaques	Type I/narrow filaments: Consist of a single protofilament with a helical twist with a crossover distance ~100 nm, width of 8 nm (min) and 13 nm (max) Type II/wide filaments: Consist of a pair of identical protofilament of type I, with a helical twist with crossover distance of ~140 nm and a width of 8 nm (min) and 26 nm (max)	CBD Fold: Identified by Zhang and colleagues, consisting of residues 274–380 of 4R tau (last residue of R1 repeat, all R2, R3, and R4 repeats, and 12 amino acids after R4 repeat). Eleven β-strands form a four-layered structure containing a hydrophilic cavity	4R
Frontotemporal dementia with parkinsonism linked to chromosome 17 (FTDP-17) (67)	Primary	Massive neuronal and glial tau deposits	Tau protofilament is an 11–12 nm diameter tubule	Not yet resolved	4R, 3R, or 3R/4R
Progressive supranuclear palsy (PSP) (68)	Primary	Tau-positive NFTs, neuropil threads and tau deposits in astrocytes (tufted astrocytes and oligodendroglial coiled bodies)	13–14 nm diameter straight tubules, two pair along longitudinal axes, show periodic narrowing at 220–400 nm and diameters of 22–24 nm (max) and 11–12 nm (min)	Not yet resolved	4R
Primary age-related tauopathy (PART) (69)	Primary	NFTs	Tau fibrils display a typical PHF morphology	Not yet resolved	3R/4R
Argyrophilic grain disease (AGD) (70–72)	Primary	Argyrophilic grains, oligodendrocytic coiled bodies and pre-neurofibrillary tangles	9–18 nm diameter straight filaments or 25 nm diameter tubules	Not yet resolved	4R

### Characteristics of Tau Aggregates

**Figure 2:**
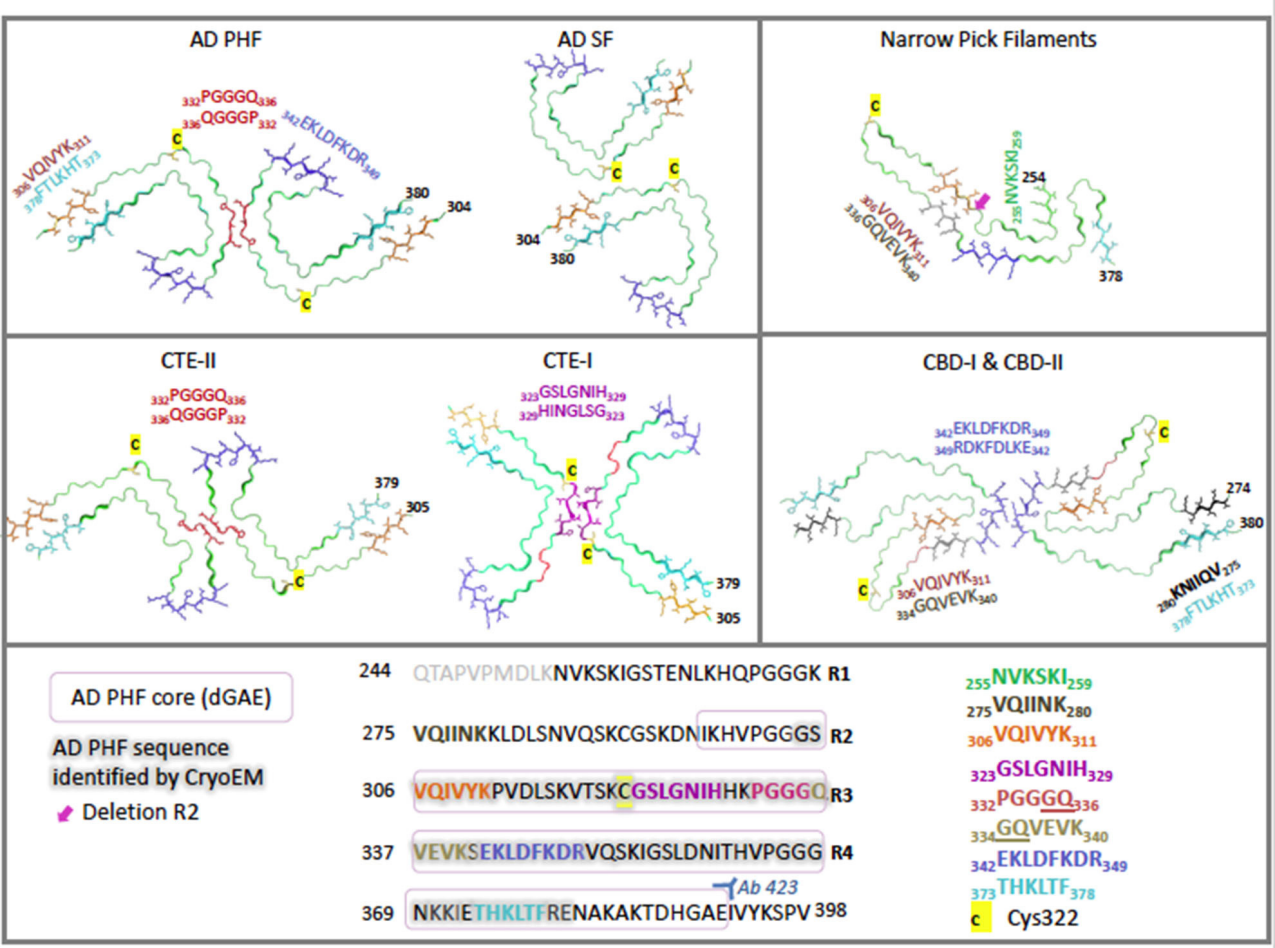
Cryo-electron microscopy structures of *ex-vivo* filaments from AD (6hre, 6hrf), PiD (6gx5), CTE (6nwq, 6nwp), and CBD (6tjx) depicting a single layer slice through the filament with a view down the filament axis using cartoon ribbons in Pymol (61). Key regions are highlighted in color as stick representations. Structures have been arranged to highlight comparisons of key region positions. The primary sequence is shown below, highlighted in the same colors for each region. Cys322 is highlighted in yellow as a stick representation. Pink box signifies AD PHF-core dGAE sequence 297–391. Gray outline signifies region resolved by CryoEM for AD PHF. Blue 342–349 is found at the β-helical bend in AD PHF and SF as well as CTE-I and -II, but is found at the dimer interface for CBD-I and -II and at a kink in PiD. Red 332–336 is found at the dimer interface for AD PHF and for CTE-II interface, while purple 323–329 is found at the dimer interface for CTE-I. Orange 306–311 (PHF6) interdigitates with cyan 373–378 across the sheets between the N- and C- terminal ends of the core in AD PHF, AD SF and CTE-I and II. However, in PiD and CBD-I and II, 306–311 interdigitates with 336–340. In the recently solved CBD structures, black 275–280 (PHF6*) interdigitates with cyan 373–378. Green is found at the N-terminus of PiD. Pink arrow points to the absence of R2 in PiD narrow filament structure. AD: (62, 63) PiD (64); CTE: (65); CBD: (66).

For each tauopathy, revealing the unique molecular signatures for the disease-specific fibrils is a vital step toward understanding key assembly mechanisms and interactions. Recent cryo-electron microscopy (cryo-EM) studies have revealed the atomic structure of the tau fibrils associated with AD, PiD, CTE, and CBD. In this section we explore the similarities and differences between the molecular structures of these aggregates (Figure 2).

#### Alzheimer's Disease (AD)

AD is the leading cause of dementia and is characterized by the presence of extracellular amyloid plaques, composed of Aβ peptide, and intracellular neurofibrillary tangles (NFTs), composed of tau (74–76). The accumulation of these pathological hallmarks causes progressive neurodegeneration in the neocortex, hippocampus and cingulate gyrus, leading to cognitive decline (77).

NFTs are composed of two types of fibrils: paired helical filaments (PHFs) and straight filaments (SFs) (49, 78–80). In AD, PHFs predominate (90%), whereas filaments in PiD are predominately SFs (95%). Initial proteolytic cleavage and antibody labeling studies established that PHFs isolated from AD brain tissue comprise of a core that is resistant to a broad-spectrum exo-protease (Pronase), which is covered with a Pronase-sensitive “fuzzy coat” (74, 81). Pronase treatment removes the “fuzzy coat” leaving the intact PHF-core, which reacts with monoclonal antibody 423 (mAb 423) that recognizes tau truncated at E391 (82). These experiments confirmed that tau is the major structural component of the PHF core (83–85). Negative-stain electron microscopy further revealed that the core comprises of a double helical stack of C-shaped subunits, with the N- and C-terminal regions projecting away from the core in a disordered manner to form the “fuzzy coat” (86).

In a recent cryo-EM study, Fitzpatrick and colleagues solved the structure of the shared common structural core of PHFs and SFs, consisting of residues V306—F378, indicating the inclusion of R3 and R4 repeats plus an additional 10 amino acids at the C-terminus (62). This allows for the incorporation of all six tau isoforms. Labeling with an R2-specific epitope antibody (anti-4R, raised against V275-C291 of R2) was used to suggest the absence of R2 from the core, although R2 sequences were identified in core PHF characterized biochemically (84). The core structure is an in-register parallel β-sheet formed by a β-bend and a β-helix motif linked by β-strands, which forms the previously identified C-shape (termed Alzheimer's fold) (Figure 2). In addition, these findings have confirmed the presence of the hexapeptide, ^306^VQIVYK^311^ (PHF6) in the core of AD filaments, located in the N-terminal part of the cross-β structure and that packs through a heterotypic, non-staggered interface with the opposing residues ^373^THKLTF^378^ (Figure 2). PHF6 has been considered essential for tau self-assembly (87, 88). PHFs and SFs are formed from two identical protofilaments that differ in the way they are packed, displaying ultrastructural polymorphism (62); PHF protofilaments are paired base-to-base, whereas SF protofilaments are paired back-to-base (Figure 2).

#### Pick's Disease (PiD)

PiD, an uncommon subtype of FTD, is a neurodegenerative disorder characterized by the presence of tau-positive filaments, contained in lesions originally described as Pick's bodies found throughout the limbic and neocortical regions. PiD presents clinically with a combination of aphasia and behavioral abnormalities. The most prevalent FTD syndrome is behavioral-variant FTD (bvFTD) and is characterized initially by personality changes and impairment in judgement in the absence of memory impairment. The broader class of frontotemporal lobar degeneration disorders includes primary progressive aphasia, corticobasal syndrome, and a Parkinsonian syndrome (89).

Cryo-EM distinguished narrow and wide Pick filaments (NPFs and WPFs, respectively), with NPFs being the primary filament observed (64). NFPs are composed of a single protofilament with a Pick tau filament fold (termed the Pick fold) (Figure 2). Whereas, the WPFs are seen to be two NPF protofilaments interacting at their distal tips. As in AD, these filaments are composed of a Pronase-resistant core surrounded by a “fuzzy coat” of disordered N- and C-terminal projections, which are removed after Pronase treatment. The Pick fold encompasses the core region, which consists of residues K254—F378 of 3R tau, including the C-terminal part of R1, all of R3 and R4, and 10 amino acids after R4. This confirmed the selective incorporation of 3R tau only in Pick body filaments. The core structure consists of 9 β-strands arranged into four cross-β packing stacks and connected by turns and arcs, which creates an elongated structure that is distinct from the C-shaped sub-structure of protofilaments seen in AD (Figure 2) (64).

#### Chronic Traumatic Encephalopathy (CTE)

CTE is a neurodegenerative tauopathy associated with repeated head trauma, identified in former participants of contact sports (boxing, American football, and soccer), ex-military personnel, and victims of severe physical abuse (90–93). CTE is characterized by an abundance of hyperphosphorylated tau protein within NFTs and glial tangles, with tau aggregates forming in cortical layers II and III (94–97).

Falcon and colleagues found two novel filament structures in CTE brain tissue (termed CTE type I and CTE type II filaments), which are ultrastructural polymorphs that share protofilaments, but with a different protofilament interface (Figure 2). In addition to these, they found a small fraction of filaments that were identical to AD PHFs (65). With the use of antibody labeling, it has been shown that all six tau isoforms in a hyperphosphorylated state are incorporated into the protofilaments similar to AD (76). The core of the protofilaments consists of residues K274-R379 of R3 tau and S305-R379 of R4 tau to form a C-shaped conformation, termed the CTE fold, which resembles the Alzheimer's fold (Figure 2). However, due to a different β-strand packing in the β-helix motif and a hydrophobic cavity, absent in AD filaments, CTE protofilaments have a more open C-shape conformation than seen in AD (see Figure 2). The hydrophobic cavity contains an additional density that is not connected to the density of the tau molecules, suggesting it is not covalently linked and does not appear to be a post-translational modification (PTM) (65). The authors suggest candidate molecules might be non-polar sterols and sterol derivatives, or fatty acids. The hydrophobic cavity containing the additional density has highlighted the structural differences seen in tauopathy filaments, as well as identifying a potentially important step in the pathogenesis of CTE. With the presence of tau inclusions around blood vessels in CTE, the authors suggest the additional density represents cofactors that may be involved in the initiation of tau aggregation in CTE, which may enter the brain after head trauma or other mechanisms (65).

#### Corticobasal Degeneration (CBD)

CBD is a neurodegenerative tauopathy characterized by neuronal and glial deposits of hyperphosphorylated tau throughout the somatosensory, premotor, supplementary motor cortices, basal ganglia and brainstem (98). Patients present with motor and cognitive decline, including corticobasal syndrome, Richardson syndrome, and Alzheimer's-like dementia (99).

Zhang and colleagues have revealed two unique filaments that comprise the neuronal and glial deposits of CBD, termed type I (narrow) and type II (wide) CBD filaments (66). Type I CBD filaments are composed of a single protofilament with a helical twist that adopts a previously unknown four-layered fold, while type II CBD filaments consist of a pair of identical protofilaments of type I, exhibiting helical twists and related by mirror symmetry. Type I and II filaments contain a common protofilament, consisting of a core structure composed of residues K274-E380, which covers the last residue of R1, all of R2-R4, and 12 amino acids after R4. The core consists of 11 β-strands that are connected by turns and arcs to form a previously unknown four-layered structure, termed the CBD fold (Figure 2). Like the CTE fold, the CBD cryo-EM map contains additional density that the authors suggest may be composed of non-proteinaceous, polyanionic molecules. Unlike CTE, the additional density in CBD is contained in a hydrophilic cavity. The authors have suggested that these molecules may enter the brain from the periphery and aid the formation of the CBD fold and filaments, as in the hypothesis of Falcon and colleagues for CTE filament assembly (65, 66).

Identifying the different conformations of tau folds in these different diseases has been a pivotal step in our understanding of tau aggregates and the important interactions involved in tau self-assembly. These structures have demonstrated the ability of tau to form distinct molecular conformations that could account for the structural diversity of tau aggregates within the different tauopathies. The structural differences seen in tau filaments from AD and CTE indicate that tau isoform composition is not the sole determinant of conformation, and that the distinct tau structures observed are the result of precise disease-specific processes (62, 65, 100).

AD is the most prominent tauopathy, thought to account for around 60–70% cases of dementia worldwide and is expected to affect 100 million patients by 2050 (101). The remainder of this review will focus on tau assembly observed in AD and the use of *in vitro* models of tau aggregation. However, it is important to consider the potential significance and relevance of tau self-assembly findings in AD research for other tauopathies.

### Tau Self-Assembly in AD

**Figure 3:**
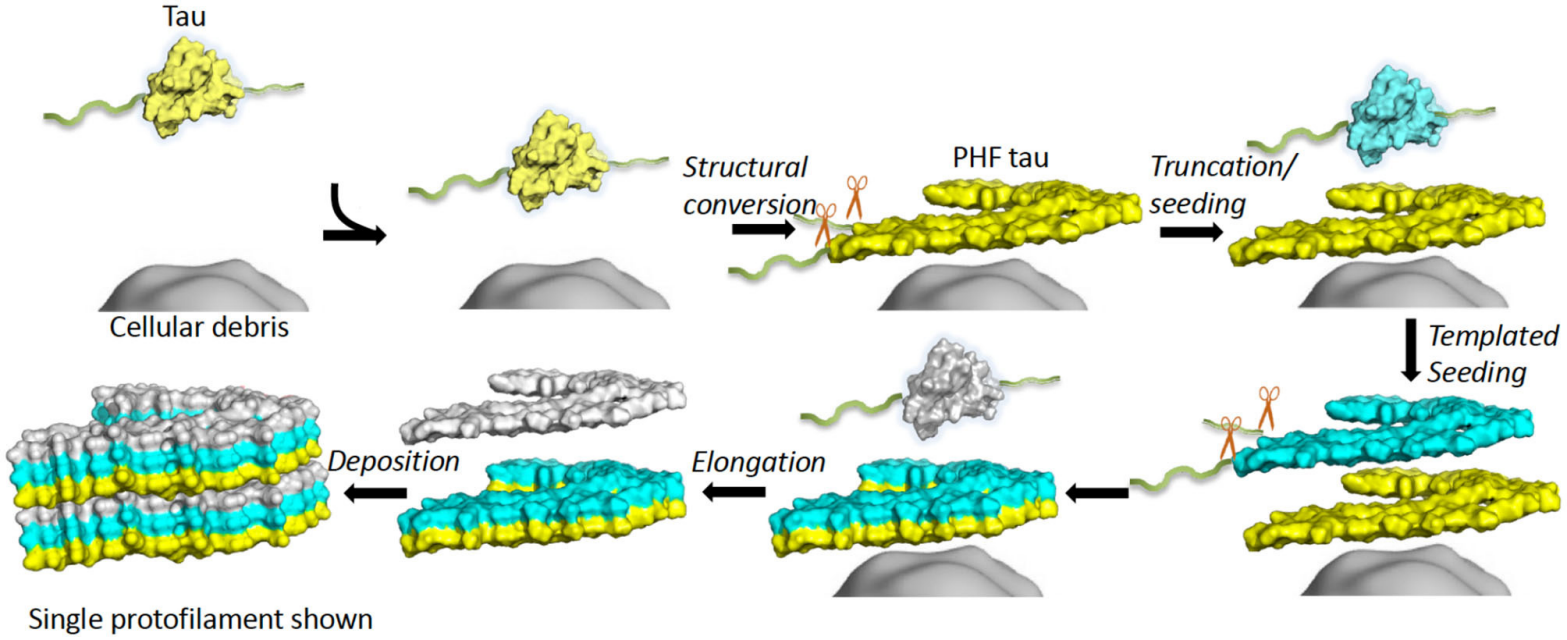
*In vitro* tau aggregation by autocatalytic propagation. Native tau misfolding (yellow) is triggered by unknown cellular components (gray surface) initiating a structural conversion. This misfolded tau (yellow) acts as a seed that binds full-length, native tau (cyan) and converts the repeat domain into a proteolytically stable conformational replicate (cyan). This process repeats (white), leading to formation of elongated fibrils (6 layers shown). Excess regions of tau outside the core (fuzzy coat) are removed prior to or during conversion or after assembly (exact details unknown). The structure of a single protofilament is shown for simplicity, although dimerization is an important step for AD PHF formation. Single chain layers are shown in yellow, cyan and white. The morphological detail is created using a single layer and chain of 6hre.pdb as a template and Pymol was used to prepare a surface representation (61).

Full-length recombinant tau shows very little intrinsic tendency to aggregate *in vitro*, due to its hydrophilic nature and absence of PTMs that may promote aggregation (102, 103). Our understanding of tau aggregation has come mostly from *in vitro* studies using tau models, in which exogenous additives are required to initiate tau aggregation, or fragments of tau that can spontaneously aggregate. Additives include polyanionic factors, such as heparin or RNA (104–106), and arachidonic acid (107). Early studies demonstrated that pathological tau-tau binding through the repeat domain instigates the formation of the proteolytically stable structure of the PHF core (108, 109). This process exhibits a “prion-like” characteristic, whereby aggregation of tau seems to be auto-catalytic and self-propagating (110). Tau protein undergoes repeated binding and proteolytic digestion cycles, leading to the accumulation of a PHF-core tau fragment at the expense of native tau. The accumulating fragment lacks the N-terminus and terminates at the C-terminus at E391 (108) (Figure 3). Key regions that drive filament formation are thought to be the hexapeptides ^306^VQIVYK^311^ (PHF6) and ^275^VQIINK^280^ (PHF6^*^), (highlighted in Figure 1) (87, 88). PHF6 has been suggested as essential for tau self-assembly (87, 111) and cryo-EM has revealed its presence in the core region, where it packs through a heterotypic, non-staggered interface with ^373^THKLTF^378^ (62) (Figure 2). However, it has been suggested that PHF6^*^ is a more powerful driver of 4R tau aggregation and a superior target for inhibiting assembly of 4R tau (112).

It is not known what initiates the self-assembly of tau protein *in vivo*. Lipofuscin deposits have been suggested as a substrate that might capture tau and induce a stable conformational change in the protein. Lipofuscin is composed of undigested products of mitochondrial turnover as a result of a dysfunctional endosomal-lysosomal pathway (109, 113, 114). Alternatively, tau on its own, either as oligomers or short fibrils, can initiate or seed the recruitment of monomers in tau self-assembly (115, 116). This process is discussed later in this review.

The events or factors that initiate the process of autocatalytic aggregation of tau protein remain unclear. No differences in the primary amino acid sequence have been identified in the tau fragment extracted from PHF preparations, which indicates that any pathological processing of tau associated with PHF assembly must be induced by conformational or post-translational changes in the protein (75, 117, 118).

Tau exists as a natively unfolded protein and is subject to many PTMs, such as phosphorylation and truncation, which affect its biological function. Nonetheless, aberrant PTMs may be associated with preventing tau function by inhibiting tau-tubulin binding, while promoting pathological tau self-assembly (119–122). Understanding these PTMs is extremely important when studying the key initiation mechanism of tau self-assembly and are therefore important when considering reliable tau models.

#### Phosphorylation

Tau is a phosphoprotein and exists in a dynamic equilibrium between a microtubule-bound and -free state, controlled by the extent of its phosphorylation. Tau contains up to 85 potential phosphorylation sites, with the main kinases responsible for tau phosphorylation identified as GSK3β, CDK2, CDK5, CaMKII, and PKA. The major phosphatases, that dephosphorylate tau include PP2A, PP2B, PP2C, with PP2A considered the main phosphatase (123–125).

PHFs from AD brains were shown to contain all six isoforms in a hyperphosphorylated state (84) and PHF-tau contains 4–5-fold more phosphorylated residues than tau from healthy control brains, taken by some to indicate an association between hyperphosphorylation of tau with tau assembly in AD (126, 127). In conjunction with this, higher levels of extracellular hyperphosphorylated tau are measured in patients with AD (128). Hyperphosphorylation of tau may drive some disease-relevant features such as a lower binding affinity for microtubules; reduced biological activity; mis-localization within the cell; increased ability to aggregate into PHFs; and to contribute to neuronal death (49, 129–138). It has been argued that tau phosphorylation could be the trigger of its self-assembly, with an increase in tau phosphorylation observed prior to the formation of tau aggregates (139–143). On the other hand, biochemical studies of tau accumulation in the course of disease do not suggest the existence of a soluble pool of free phosphorylated tau prior to aggregation (144), and indeed the appearance of soluble forms of hyperphosphorylated tau becomes measurable in the cortex only at advanced stages of pathology (56). Other studies have suggested that it is not the overall phosphorylation state of tau that may trigger aggregation, but the phosphorylation of specific sites. Combined phosphorylation of S202/T205/S208 sites in the AT8 epitope (shown in Figure 1), with the lack of phosphorylation at S262, contributes to filament formation (145). In contrast, Zhou and colleagues observed a dramatic increase in the phosphorylation sites, including S262, in association with the formation of high molecular weight oligomers of tau in AD brains (146). In addition, Tepper and colleagues suggested that phosphorylation of tau in cells promotes oligomer formation but not filaments (147). Alternatively, phosphorylation may be secondary to formation of oligomers and consequent mis-sorting of tau (110).

The involvement of tau phosphorylation in tau assembly is supported by the observation that there is a 50% reduction in the prominent phosphatase, PP2A, observed in AD brains (124). Dephosphorylation of PHFs isolated from AD brains results in their dissociation and disaggregation (148), as well as restoring normal tau function to promote assembly of microtubules and abolishing its pathological self-assembly (131, 149). These studies support an association between hyperphosphorylated tau and its self-assembly into PHFs. However, other groups have shown conflicting results. Initial studies of the PHF core revealed that, after Pronase treatment, PHFs were not recognized by phosphorylation-dependent antibodies (74, 81). Other studies have shown that only a minor proportion of PHF-tau is either full-length or hyperphosphorylated (<5%) (150) and tau species isolated from the proteolytically stable PHF-core are not phosphorylated (151). This suggests that the sites of tau phosphorylated in the PHFs are located only in the “fuzzy coat” and are not structural components of the PHF-core. This has been further supported by Arkhamia and colleagues, who showed that phosphorylation occurs largely in the “fuzzy coat” region of CBD and AD filaments using mass spectrometry (152). Furthermore, the structural core revealed by cryo-EM comprises regions downstream of key phosphorylation sites such as the AT8 epitope (153–155) (Figure 2). Additional studies have questioned the role of phosphorylated tau in the mechanism of tau self-assembly. Studies have implied that hyperphosphorylation of tau inhibits tau-tau binding (109, 156), inhibits its aggregation (157), and is not sufficient for filament propagation in cellular models (158, 159).

#### Truncation

Tau is sensitive to proteolytic digestion that is facilitated by a group of proteases. Tau is a substrate for a variety of proteases, such as caspase (160), calpain (161), cathepsin (162), and thrombin (163). As previously stated, initial studies identified truncated tau as the structural component of the Pronase-resistant PHF core (74, 81). Truncation of tau protein can act as a driver of tau self-assembly in the process of autocatalytic propagation by removal of occluding N- and C-terminal domains of the molecule (Figure 3) (108, 110). As a natively unfolded protein, tau has little tendency to aggregate in physiological conditions (103). However, there is evidence that tau can change its conformation into a “paperclip” loop, by folding its N- and C-terminal regions back onto the microtubule-binding repeats, thought to inhibit or protect against pathological aggregation (164). Tau fragments truncated at both N- and C-termini that contain the microtubule-binding repeats are more prone to form aggregates, probably because the protective “paperclip” loop has been disrupted (165). It has been suggested that truncated tau can facilitate the assembly of full-length tau for fibril formation and is the driving force behind NFT formation (47, 160, 166, 167). The self-assembly of truncated tau observed with the *in vitro* model dGAE form fibrils that structurally resemble PHFs in AD without inducers (168, 169); dGAE is one of the PHF-core tau species isolated from AD brain tissue (74, 84).

PHF-core tau isolated from Pronase-treated PHF preparations from AD brain comprises a number of different fragments encompassing residues 297–391 and 264–359 from the repeats R2–R4 and repeats (R1–R3) of the 4-repeat isoform, and residues 266–391 of the 3-repeat isoform (74, 82, 83). The 296-390 fragment binds full-length 4-repeat tau with high affinity (156) and generates the species terminating at E391 *in vitro* (108). Tau C-terminally truncated at E391 is found in early amorphous deposits in AD brain tissues and the epitope becomes occluded as filaments form (170). Tau truncated at E391 was identified in PHFs isolated from AD brains after-Pronase treatment using mAb 423, which recognizes the C-terminal E391 in PHFs, but not normal adult tau proteins (85, 171). The E391 truncation and truncations at homologous positions in other isoforms, therefore represent the proteolytically stable footprint of the high affinity core-tau aggregation domain (82).

Tau truncated at D421 is produced by caspase activity, which is elevated in AD brains (172, 173). It has been shown that Aβ (1–16, 23–48) peptide exposure can trigger caspase cleavage of tau to produce the N-terminal tau 1–421 fragment, which assembles more readily into filaments than full-length tau. This study suggests a possible link between Aβ, induction of tau PTMs and the initiation of tau self-assembly into filaments (160). In addition, studies have indicated that caspase-mediated truncation of tau at D421 may precede hyperphosphorylation and aggregate formation in AD, PiD and PSP, which may be an important step in understanding tau self-assembly for tauopathies (174–177). Studies have also demonstrated that tau truncated at D421 may become an effector of apoptosis, further linking pathological tau to neurodegeneration (178, 179).

On the other hand, an *in vivo* study using the P301S transgenic mouse model demonstrated that hyperphosphorylation precedes the truncation of tau at D421 and that truncation occurs after filament formation (180). This suggests that this particular truncation is not necessary for filament formation. Further studies indicate that phosphorylation and N-terminal truncation, but not C-terminal truncation, are more significant in the formation of oligomeric tau species (146). This further demonstrates the complexity of tau self-assembly and highlights the gaps in our understanding of the initial steps in the formation of aggregates. Identifying an *in vitro* model that can accurately replicate the pathological processing of tau in AD brains will greatly assist in studies on tau self-assembly, as well as other important aspects of tau pathology, such as its propagation throughout the brain.

### Trans-Cellular Propagation of Tau and Seeding

If tau pathology were restricted to affected neurons, then the damage induced by tau would be self-limiting. As discussed earlier, propagation of tau is thought to be through a prion-like mechanism, which utilizes an initial seed to recruit normal monomeric tau and is able to facilitate autocatalytic amplification (110). The initial seed could be undigested cellular components, such as lipofuscin deposits (109), or it could be tau in an oligomeric state. Recent studies have supported the proposal that tau itself can undergo active cell-to-cell (trans-cellular) transfer and initiate the propagation of tau aggregates in previously unaffected neurons, whereby it seeds further tau aggregation (181, 182). Understanding the mechanism whereby pathological tau can spread throughout the brain is a major focus for dementia research and offers the potential of being an important therapeutic target for attenuating the progression of tauopathies.

Initial studies observed the formation of intracellular tau aggregates after tau was applied extracellularly, demonstrating the ability of tau to seed assembly in adjacent cells *in vitro* (183) and *in vivo* (184). It was further shown that intracellular tau aggregates are released and taken-up by co-cultured cells, whereby the internalized aggregates induced assembly of intracellular tau and promoted fibrillization, demonstrating prion-like propagation of tau (115, 181, 182). Additional studies have shown that the trans-cellular propagation of tau is facilitated by synaptic connections between neurons (trans-synaptic propagation) that seems to be stimulated by depolarization during neuronal activity, which can accelerate the spread of tauopathy (185–191). The spread of tau through trans-synaptic propagation via specifically connected neurons may explain the stereotypical staging of tau pathology seen in AD (192). However, the mechanisms that facilitate tau intercellular propagation through secretion and uptake are not fully understood. Several studies indicate that tau secretion is enabled via vesicles known as exosomes (193–196) and ectosomes (188). Microglial cells may also promote tau propagation through exosome-dependent mechanisms (197). In contrast to these findings, a study indicated the secretion of tau is an exosome-independent process, requiring heat shock cognate 70, chaperone DnaJ and synaptosomal associated protein 23 (198). The secreted pathological tau is required to undergo cellular uptake to gain entry into neighboring cells, which has been linked to multiple cellular uptake mechanisms. These include receptor-mediated endocytosis (199), dynamin-driven endocytosis (187), and actin-dependent proteoglycan-mediated micropinocytosis (200).

In addition to the mechanisms that govern intercellular propagation, it is becoming increasingly important to consider the species of tau that is responsible for initiating seeding. Proteolytically stable oligomers have been the mostly widely documented species responsible for intercellular self-propagating tau and have the greatest seeding activity for initiating the self-assembly cascade of tau in unaffected brain regions (115, 183, 184, 187, 201). Recent work has shown that the dGAE fragment in soluble form can be internalized into neuronal cells and can recruit endogenous tau (202). There is also some evidence for fibril-induced tau propagation (203, 204). Nonetheless, oligomeric tau is still thought to be the key species that gives tau pathology its “prion-like” characteristic.

Recognizing the self-propagating nature of tau and the seeding abilities of different tau species in tauopathies has opened up new possibilities for therapeutic targets and interventions for the treatment of tauopathies. The anti-tau antibody infusions and small molecules to disrupt the propagating mechanism of tauopathies are attractive therapeutic strategies. These therapeutic targets will be discussed later in the review.

Although NFTs are considered histopathological hallmarks of AD, there is increasing evidence that the cytotoxicity associated with tauopathies may not be as a result of these larger aggregates. In addition to trans-cellular propagation, oligomers are associated with the molecular dysfunctions that underlie neurodegeneration and the cognitive decline seen in patients. *In vivo* tau-overexpression studies have demonstrated neuronal loss, behavioral abnormalities, and synaptic dysfunction occurring in the absence of NFTs (205–212). These studies indicate a pathological role for pre-tangle tau species in the dysfunction of neurons. This has been further supported by evidence that injection of tau oligomers into wild-type mice induces memory impairment, synaptic loss, and mitochondrial dysfunction, that are not seen with monomer or fibril injections (213). In addition, following a decrease in oligomeric tau levels, improved cognitive function suggests the potential of anti-tau antibody infusions as a therapeutic approach (214). In response to these findings, it has been proposed that mature tau fibrils could be a protective response to absorb the more toxic oligomeric species, eventually inducing their clearance via protease activity or autophagy (215, 216). However, the oligomers formed through the repeat domain are proteolytically stable and hence resist proteolytic and autophagic activity, and are therefore able to propagate between neurons and induce cytotoxicity. Tau-induced neurodegeneration may be due to an increase in oligomers rather than due to the accumulation of PHF in NFTs. This concept has been proposed for other neurodegenerative diseases, but still remains to be validated with respect to tau and AD (217).

Investigating the molecular mechanisms underlying protein self-assembly has been an important focus for further understanding of neurodegeneration and identifying therapeutic targets. Generating a reliable *in vitro* model that can accurately replicate the protein self-assembly and associated cytotoxicity that occurs in tauopathies will further our understanding of the underlying principles of aggregation and the potential for therapeutic targets. However, it presents a huge challenge to create an *in vitro* model that will effectively replicate pathological processing of tau that occurs *in vivo*, whilst also modeling other aspects of interest, such as the production of representative oligomeric species.

### Models of Tau Aggregation

**Figure 4:**
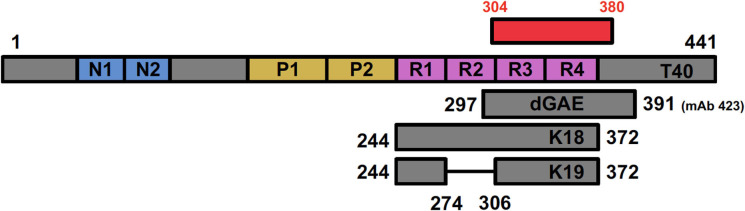
Positioning of tau fragments used as models for studying tau biology showing full-length T40 in relation to the AD cryo-EM derived structure (shown in red). dGAE covers I297—E391, incorporating a C-terminal portion of R2, R3, and R4 (shown in pink). The C-terminal E391, recognized by mAb 423, is absent in dGA, a fragment ending in A390. K18 (Q244—E372) covers all four repeat domains; K19 covers the same region but lacks residues 275–305 (R2) encoded by exon 10 in 4R isoform.

Brain-derived materials are inherently variable, and variations are difficult to control. Hence, there is a need for an *in vitro* system using synthetic tau that can recapitulate the pathological self-assembly of tau observed in the brains of tauopathy patients. Due to its hydrophilic nature, recombinant tau shows very little tendency to aggregate, making it a difficult protein for study of its aggregation and cytotoxicity mechanisms (102). Thus, multiple tau models have been developed that exhibit a higher tendency to aggregate and enable effective *in vitro* research. Here we discuss some of these models, summarized in Figure 4.

### Full-Length Tau (T40, 2N4R)

The largest tau isoform, also known as full-length tau or T40 (441 residues), incorporates both N-terminal inserts and four microtubule-binding repeats (2N4R tau: Figure 4). The full-length tau *in vitro* model will be referred to as T40 for this section of the review. This isoform has been used extensively to study the role of tau in disease. Tagging T40 with diverse fluorescent proteins (GFP, YFP, and CFP) has been a useful tool to visualize tau aggregation *in vitro* and further our knowledge of tau assembly (153, 218). Frost and colleagues used fluorescently tagged endogenously expressed T40 in their studies to demonstrate tau propagation and seeding, as summarized earlier. They showed that tau-YFP does not readily aggregate within the cell but, when they introduced tau as a pre-assembled aggregate into the cell, it induced fibrilization of the tau-YFP (183). Groups have been able to develop cell-based models that can monitor and quantify tau assembly by utilizing fluorescence resonance energy transfer and biomolecular fluorescence complementation technology using T40 (154). Using these techniques, groups were able to achieve spatial and temporal resolution of tau aggregation in living cells following hyperphosphorylation (219), and demonstrating the role of GSK3β and caspase cleavage in intermolecular association of tau (220, 221).

Many investigators have used T40, either as a monomer or having induced filament assembly by the addition of cofactors, such as heparin (104, 105, 155), RNA (106), or fatty acids (107). T40 filaments resemble those found in AD brains. These cofactors enhance tau aggregation by inducing a conformational change from mostly random coil to β-sheet structure in the microtubule-binding domain containing the hexapeptide motifs thought to be necessary for assembly (87, 88, 222, 223). Heparin-induced filaments have become an important tool used to study pathological tau aggregation, such as investigating the effects of *MAPT* mutations on tau assembly (224, 225) and investigating potential aggregation inhibitors (226–228). They have provided a platform to characterize the progressive fibrilization of tau, through monomeric aggregation into intermediate species and ultimately into filaments (229). In addition, heparin-induced aggregates of T40 were used to support the role of PHF6^*^ hexapeptide (^275^VQIINK^280^) as a potent driver of aggregation and seeding for full-length tau, suggesting that it is a superior target for inhibition when compared to the PHF6 hexapeptide (112). Crespo and colleagues have used heparin-induced filaments to produce an assay, which they claim to be highly reproducible and closely mimicking *in vivo* tau misfolding and aggregation (230). They suggest that the assay will enable mechanistic studies on tau pathogenesis and can be utilized for evaluating candidate drugs during screening processes.

These studies illustrate the importance of full-length tau as a tool in understanding of both physiological and pathological tau. However, T40 may not be a reliable model for AD research. Evidence shows that heparin-induced tau has reduced seeding activity compared to tau from the brains of mice expressing human P301S tau (231). Additional studies have shown structural differences in tau filaments seeded using tau from AD brains and recombinant tau assembled with heparin (159). Recently, Fichou and colleagues reported that heparin-induced tau filaments do not reproduce the main structural features seen in filaments from AD brains, such as the C-shape Alzheimer's fold and the anti-parallel β-sheet structure between 303–347 and 349–378 (100). Moreover, cryo-EM revealed that heparin-induced filaments of 4R (full-length) tau are polymorphic, adopting at least four different conformations, with 3R tau adopting a conformation of its own (232). All of these conformations are structurally distinct, consisting of an ordered core structure, which is very different to those characterized using cryo-EM of filaments from AD, PiD, CTE, and CBD. The studies also revealed that these filaments lack essential features of AD PHFs, such as the interface between PHF6 and its opposing residues 373–378. These findings indicate that heparin-induced filaments of T40 tau are both structurally heterogeneous and distinct from those found in the disease brain, challenging their reliability for use in a tau self-assembly model or as an assay for screening potential inhibitors. As noted above, heparin-induced filaments from full-length tau were used as a basis for inferring the importance of PHF6^*^ in assembly (112). Recent cryo-EM structures of the heparin-induced filaments indicate that the PHF6^*^ region is located in the cryo-EM core of T40 filaments forming a contiguous β-strand, whereas it not found within the core of AD filaments. It is however visible within the core of the recent cryo-EM structure for CBD filaments at the far N-terminal end (residues 274–280), so it may yet be resolved in more detailed PHF structures (Figure 2). This illustrates a potential disadvantage of the heparin-induced T40 model for investigating tau self-assembly. This model produces filament structures that are distinct from those so far characterized from tauopathies and may have a different mechanism of self-assembly and highlighting factors that are not applicable for tauopathies.

Nevertheless, it is important to consider what we can learn from these heparin-induced filaments. Even though there are structural differences between such filaments and tauopathy filaments, they do exhibit similarities. They are composed of tau molecules that form cross-β structures through parallel stacking of β-strands along the helical axis. The β-strands contain short loop regions and the filament structures share a common pattern of β-strand formation (232). Studying the formation of these filaments may shed light on key assembly processes, or they could be more relevant for other tauopathy filaments that have not been solved yet, such as PSP. In addition, cofactor-induced aggregates spontaneously disassemble into monomers when the cofactor is removed (233). The authors of these studies have proposed that unknown cofactors may play a role in tau self-assembly in tauopathies and these could provide a potential therapeutic target.

Overall, T40 has been used extensively to model tau assembly. It has been extremely useful in uncovering the role of tau, both physiologically and in pathology. The use of heparin to induce aggregation of tau into filaments that may have some resemblance to disease-like filaments has made it an attractive model when investigating tau self-assembly. Given the recent evidence of structural dissimilarities from native filaments, T40 heparin-induced filaments should be used with caution in structure-based studies for drug design and antibody development, where structural accuracy is essential. This does not preclude the use of T40 in assembly assays, but rather reveals that the unique structures of heparin-induced T40 filaments differ from the pathologic filaments. The use of full-length tau in the generation of filaments should still be open to further investigation, using a range of biophysical techniques to determine the mechanism of initiation of aggregation. Future research may unveil a novel mechanism for induction of aggregation of T40 which may make it a more suitable candidate for the investigation of tau self-assembly.

### Repeat Domain Models (K18/K19)

Since the microtubule-binding repeats domain were shown to compose the core region of PHFs and facilitate the pathological tau-tau binding (74), it has been common practice to use tau fragments that only comprise of the microtubule-binding domain. Some groups simply refer to them as the microtubule-binding region (MTBR) and specify whether they use the 3R or 4R isoforms. Others use two model peptides, termed K18 and K19. K18 represents all four-repeat domain and contains residues 244–372, while K19 is similar to K18 but lacks the second repeat (missing residues 275–305) absent from 3R tau isoforms (Figure 4) (234, 235). As stated previously, tau fragments including MTBR and truncated at both N- and C-termini are more prone to form aggregates than full-length tau (165) and aggregation can be further stimulated by including mutations or adding cofactors, such as heparin (102, 236). They produce filaments that resemble AD PHFs in terms of fiber morphology and β-sheet content (87, 88, 227), which provided the basis for using MTBR in tau assembly studies.

As mentioned previously, the Diamond group have used full-length tau in their study on the propagation and seeding of tau (183). The full-length tau did not aggregate spontaneously in cells, so they utilized a MTBR fragment similar to K18, corresponding to residues 243–375. They were able to prepare *in vitro* aggregates of the MTBR fragment with arachidonic acid, which readily entered cells and induced aggregation of intracellular T40-YFP. This demonstrated that T40 does not readily aggregate in cells, but internalized MTBR aggregates were able to induce fibrilization of the intracellular T40-YFP. These studies also used the expression of mutant forms of the MTBR models to further our understanding of tau propagation. Multiple K18 mutants, labeled with YFP or CFP, were used to demonstrate that the secretion of fibrils into the extracellular space leads to the internalization of these fibrils by other cells and the aggregation of the intracellular tau, supporting the concept of trans-cellular propagation of tau aggregates (181). The authors advocate the use of the K18 construct on the grounds that it reliably forms fibrils in cultured cells and aggregation potential can be modulated with the introduction of specific mutations. This includes the combined P301L and V337M mutant (termed LM), enabling efficient formation of intracellular aggregates and providing an effective model of tau propagation. In addition, the expression of the aggregation-competent MTBD fused to YFP was utilized to confirm the prion-like nature of tau (182). Similar to T40, filaments can be prepared using K18 and K19 with the addition of cofactors. The Mandelkow group have used the heparin-induced aggregation of K19 extensively as a model in investigating inhibitors of tau polymerization. They have utilized the K19 model to screen a library of 200,000 compounds in the search for potential inhibitors of tau aggregation, and therefore potential therapeutic agents. They were able to identify rhodamines, phenylthiazolyl-hydrazides, N-phenylamines, anthraquinones and benzothiazoles (237–239) and aminothienopyridazines as a novel class of tau assembly inhibitors (240, 241). They also utilized the K18 and K19 fragments to identify the hexapeptide motifs considered to be essential for tau aggregation, PHF6 and PHF6^*^ (87, 88).

Although these studies have proved informative in tau self-assembly studies, it is now possible to compare filaments composed of K18 and K19, with genuine filaments isolated from AD brains. The cryo-EM of PHFs has shown that the N-terminal cross-β structure is formed by the PHF6 hexapeptide, which is essential for assembly of tau (87, 88) (Figure 2). PHF6 packs against its opposing residues 373–378, which are not present in the K18 and K19 fragment because they terminate at E372 (62, 234). Thus, filaments composed of K18 and K19 fragments cannot be said to reproduce the Alzheimer's fold resolved in PHFs and SFs derived from AD brains. In addition, since K18 and K19 terminate at E372, these fragments lack the C-terminus after the MTBR, that has been shown to contribute to the core region of filaments seen in AD, PiD, CTE, and CBD (in AD this extends to residues 378, in PiD to 378, in CTE to 379, and in CBD to 380), which casts further doubt on the use of such filaments as models for assembly assays. As stated previously, the use of heparin to induce K18 and K19 filaments must be used with caution, as it has been shown that heparin-induced filaments are highly heterogenous and differ from those seen in tauopathies (232).

### dGAE Model

dGAE is a 95 amino acid truncated form of tau protein corresponding to residues 297–391 encompassing part of R2, and R3 and R4 from full-length 2N4R tau (Figures 2, 4). The fragment corresponds to one of the species isolated from the proteolytically stable AD PHF core preparations (74, 82, 84). The terminology of dGAE refers to the three C-terminal residues, glycine (G), alanine (A), and terminating with glutamate (E), E391; the initial letter corresponds to the N-terminal residue which for “d” refers to I297 (82). Tau truncated at E391 was identified as being part of the Pronase-resistant core during initial proteolytic and antibody studies, which is recognized by mAb 423 (Figure 2). PHFs isolated from AD brain without Pronase are also recognized by mAb 423, but require a higher concentration of antibody (82). mAb 423 immunoreactivity can also be revealed in intracellular tangles following treatment with formic acid in dystrophic neurites and in granulovacuolar degeneration in AD brain sections (170, 242, 243) supporting the view that C-terminal truncation at E391 is not a Pronase artifact. Rather, digestion by endogenous proteases may be one of the events leading to PHF formation and depends on a particular configuration of tau found in partially assembled precursors, and also in fully assembled PHFs. The dGAE peptide and other core peptides were proposed as representing an integral part of the stable core region in PHFs (82, 108, 170, 244, 245) and has now been confirmed as encompassing slightly shorter PHF ordered core region (304–380) resolved using cryo-EM (62) (Figure 2).

dGAE was chosen for further study because it is also similar to repeats R1/R3/R4 from the 3-repeat isoform apart from 3 residues near the N-terminus (L/I, Q/V, K/S in the 3-repeat and 4-repeat isoforms, respectively) and is therefore likely to be representative of the behavior of both isoforms (Figure 2). Studies of the assembly properties of the dGAE peptide has revealed that it has the ability to self-assemble spontaneously in physiological conditions *in vitro*, without the need for added cofactors, such as heparin. Self-assembled dGAE filaments have been shown to share characteristics of amyloid fibrils, displaying β-rich structure and giving a cross-β X-ray fiber diffraction pattern (168), as well as closely resembling PHFs in AD brains (169). Transmission electron microscopy revealed no significant variations between dGAE filaments and no significant variations between dGAE filaments and AD filaments. Atomic force microscopy also demonstrated that dGAE forms filaments that share macromolecular characteristics with PHFs from AD brains. These results suggest that dGAE functions as a consistent and reliable model for understanding pathological tau self-assembly and for screening inhibitors and understanding their mode of action.

Disulphide crosslinking between dimers was proposed to be an essential part of tau assembly and propagation (102, 246–248). Disulphide cross-linkages are formed between cysteine residues, located on tau at C291 and C322 (numbering for 2N4R tau), in which C322 exists in all tau isoforms, whereas C291 is only found in 4R tau isoforms. The assembly of filaments from dGAE was found to be enhanced in conditions unfavorable for disulphide crosslinking formation and with a variant lacking the cysteine residue all together, dGAE-C322A (168). The authors concluded that assembly of dGAE is independent of disulphide crosslinking and suggested that the formation of disulphide bonds competes with crucial conformations required for filament formation. This is at odds with previous suggestions that disulphide-linked dimer formation is an important step in filament formation, but is consistent with the structure of *ex vivo* PHFs by cryo-EM (62). This shows that the C322 residue is buried between sheets and would be inaccessible for disulphide crosslinking (62, 168) (Figure 2). This evidence suggests that dGAE provides a more accurate representation of pathological tau in AD, in comparison to the models used in previous studies (1N3R, 1N4R, and K18), which require exogenous heparin for assembly. In addition, Al-Hilaly and colleagues identified two monomeric isoforms of dGAE, one with a gel mobility of 10 kDa and the second with a mobility of 12 kD. The same doublet was seen in the proteins extracted from the core region of PHFs isolated from AD brain tissue, which first lead to the identification of truncated tau as a structural component of the PHF cores (74, 168).

The dGAE fragment has also been utilized for the investigation of tau aggregation inhibitors (TAI). dGAE, and a fragment similar to dGAE but lacking the E391 residue (dGA), were used in the study which identified the methylthioninium (MT) moiety as the first TAI (108). Since then, other groups have attempted to understand the mechanisms of MT action by using heparin-induced filaments composed of the MTBR fragments, K18 and K19 (249, 250). These studies hypothesized that the TAI activity of MT requires cysteine oxidation, which was consistent with the previous proposals stating that tau assembly was dependent on disulphide crosslinking. However, a recent study using the dGAE fragment has shown that MT acts optimally as a TAI in its reduced form as leuco-MT (LMT) rather than the oxidized form at MT^+^ (251). The evidence that LMT is the active form of MT required to inhibit pathological aggregation of tau is at odds with the proposal that its action depends on the oxidation of cysteine residues (249, 250). The ability of LMT to act as a TAI is also consistent with an earlier study showing that dGAE assembly into PHFs is independent of disulphide crosslinking. In addition, circular dichroism has revealed that heparin interacts with MT. This confounds studies aiming to understand how LMT acts, and further supports the use of dGAE for such studies (251). LMT will be discussed in more detail later in the review.

We can only compare dGAE and AD filaments directly once a structure has been resolved for dGAE through the use of cryo-EM or solid-state nuclear magnetic resonance. As previously mentioned, the filaments seen in CTE share similar morphology with AD filaments, but cryo-EM revealed subtle ultrastructural differences, which could be the case for dGAE filaments and PHFs. However, the studies to date highlight the use of dGAE as a reliable *in vitro* model able to replicate tau self-assembly in a controllable manner. It may therefore prove to have broader use as a relevant tool for the investigation of assembly mechanisms and the potential therapeutic targets.

## Alzheimer's Disease Therapies Targeting TAU Self-Assembly

The clear involvement of tau self-assembly as a driving force behind the clinical progression of AD, as well as other tauopathies, has established it as an attractive target for potential therapies. Treatments for AD are extremely limited and offer only temporary symptomatic relief based on neurotransmitter enhancement, without providing disease-modifying activity. Extensive clinical studies have resulted in over 400 failed trials since the last AD treatment was approved in 2003 (47, 252). Targeting tau self-assembly to reduce disease progression has resulted in promising results and may provide much-needed disease-modifying treatment options for AD. Here we discuss some of the treatment options targeting tau self-assembly in AD, utilizing small molecule and immunotherapy approaches.

### Tau Aggregation Inhibitors (TAI)

Low molecular weight compounds offer a promising approach for inhibiting the formation of oligomers and fibrils by blocking tau-tau interactions. The microtubule-binding domains have been shown to be responsible for the pathological tau-tau binding, but also facilitating the tau-tubulin binding required for physiological tau function (27, 108). Thus, TAIs should inhibit pathological tau-tau binding whilst not affecting the physiological tau-tubulin interaction, although both occur through the repeat domain. Potential side-effects are extremely important to consider for compounds destined for clinical trials. Wischik and colleagues demonstrated the first TAI to be MT, a diaminophenothiazine. Since then, other compounds have been shown to exhibit TAI activity, such as the potential TAIs identified by the Mandelkow research group, mentioned previously (237–239). None of these potential TAIs have yet reached clinical testing. Both the oxidized MT^+^ and reduced LMT forms of MT have shown promising results in Phase 2 and Phase 3 clinical trials, respectively.

Methylthioninium chloride (MTC, commonly known as methylene blue) was originally synthesized at the end of the nineteenth century for the treatment of malaria, and since then has had a long history of clinical applications. Importantly for AD research, Wischik and colleagues reported MTC as the first TAI, demonstrating its ability to reverse the proteolytic stability of PHFs isolated from AD brain and selectively inhibiting tau self-assembly, without affecting normal tau-tubulin interactions (108). Additional *in vitro* and *in vivo* studies have confirmed MTC as an effective TAI (245, 251, 253). As noted above, it is actually the LMT form which is the active species responsible for TAI activity in the dGAE model of pathological tau assembly. Other studies have suggested that MTC induces degradation of tau aggregates by promoting the proteasomal (254) and macroautophagic (255, 256) clearance systems. The MT moiety has also shown potential for ameliorating mitochondrial dysfunction and reducing oxidative stress, by acting as an alternative electron carrier for the electron transport chain in oxidative phosphorylation (257–264). *In vivo* studies have confirmed that both MTC and a stabilized form of LMT [LMTM, leuco-methylthioninium bis(hydromethanesulfonate)] are able to preserve cognitive function and reducing behavioral deficits associated with pathological tau aggregation in tau transgenic mouse models (253, 265, 266). In cell models, LMTM was shown to block the templated conversion of full-length tau into the truncated 12 kDa form analogous to dGAE (245). Likewise, the tau species used in the Melis and colleagues study to produce tau aggregation pathology in mice depends on the overexpression of a truncated form of tau analogous to dGAE (253).

The pharmacology of MTC is complex, existing as a redox molecule in equilibrium between its reduced LMT form and its oxidized MT species and dependent on the local environment (253). Because its redox potential is close to zero the MT moiety is able to act as an electron carrier between Complex II and Complex IV in the electron transport chain, permitting it to enhance mitochondrial function (257, 267). However, when administered as MTC, the MT moiety needs to be reduced to its uncharged LMT form to permit efficient absorption and distribution to the brain (265). As noted above, MTC is optimally effective as a TAI when converted to LMT in a reducing environment, implying that LMT is the active species required for inhibition of tau aggregation (251). Optimizing the clinical effectiveness of LMT has been a challenge in clinical trials. The MT moiety was first tested clinically in a pure, oxidized MTC form under the name Rember™ (TauRx Theraputics) in Phase 2 clinical trial in mild-to-moderate AD. This showed significant efficacy compared with placebo in terms of reduction in cognitive decline and prevention of decline in brain metabolism as shown by HMPAO-SPECT scan which measures cerebral blood flow (268). However, MTC was found to suffer from dose-linked absorption limitations (268) arising from the need for the body to convert MT^+^ to the active LMT form required for efficient absorption and distribution to the brain (265). A stabilized form of the reduced LMT was developed which permits direct administration of LMT as the mesylate salt, LMTM. LMT has recently been assigned the International Nonproprietary Name “hydromethylthionine.” LMTM retains TAI activity *in vitro* and *in vivo* (245, 253), whilst exhibiting superior pharmaceutic properties (265). Results from Phase 3 trials with LMTM have shown promising results with exposure-dependent reduction in cognitive decline and progression of brain atrophy as measured by MRI. LMTM was found to produce better outcomes when given as a monotherapy than as an add-on to symptomatic AD treatments (269, 270). However, a recent report has demonstrated that there is similar exposure-dependent activity whether LMTM is administered as a monotherapy or as an add-on, but that the overall effect was reduced by about half as add-on (271). This impairment in the activity of LMTM by symptomatic AD drugs could be reproduced in a tau transgenic mouse model for AD, and was found to depend on the brain's homeostatic responses to the activating affects that symptomatic drugs depend on for their ability to boost cognitive function temporarily (267). The concentration-dependent effects showed that LMTM requires a substantially lower dose for pharmacological activity than found for MTC (271). Currently, TauRx is undertaking a new clinical trial (LUCIDITY) which aims to confirm the treatment effects of LMTM on cognitive decline and brain atrophy in mild-to-moderate AD.

Interestingly, LMTM was found to have an almost identical exposure-response profile in a large Phase 3 clinical trial of bvFTD as that seen for AD (272). Fewer than half of bvFTD patients have tau aggregation pathology, with the remainder exhibiting pathological aggregation of TAR DNA binding protein 43, TDP-43. LMTM was found to have activity in mouse model of FTD expressing full-length human tau carrying mutations at P301S and G335D (253). The fact that LMTM has similar pharmacological activity in both AD and bvFTD raises the possibility that the subtle folding differences in the tau filament core in the two diseases may not be important for pharmacological activity for this drug. There is as yet no *in vitro* model for Pick-like tau aggregation as distinct from AD-like tau aggregation to permit examination of this question. However, this example illustrates again the importance of developing reliable and reproducible models that can be used to study distinct mechanisms of aggregation and activity of TAIs.

Curcumin is another compound that has been reported to exhibit TAI properties. Curcumin is a natural polyphenol isolated from the Indian spice turmeric, which has been reported to inhibit assembly of tau (273–275) and Aβ (276). However, curcumin has poor bioavailability and had no effect on cognition or cerebrospinal fluid biomarkers in a Phase 2 trial (277, 278). Therefore, a derivative of curcumin, PE859, has been synthesized that exhibits higher inhibitory activity against tau and Aβ assembly *in vitro*, with high absorption and penetration of the blood brain barrier. Oral administration of PE859 delays onset and progression of motor dysfunction in JNPL3 human P301L tau transgenic mouse (279). Further investigation illustrated the effects of PE859 *in vivo*, demonstrating its dual aggregation inhibitory activity and ameliorating cognitive dysfunction in the senescence-accelerated mouse prone 8 (SAMP8), which show age-related deficits in memory and learning (280). Okuda and colleagues were also able to show that PE859 is protective with respect to Aβ-cytotoxicity *in vitro*. The potential for combined anti-aggregation activity for tau and Aβ makes PE895 an attractive candidate for clinical trials.

### Immunotherapy

The potential use of immunotherapy to combat protein self-assembly was initially recognized with studies using antibodies targeting Aβ peptide, which resulted in attenuating Aβ pathology through preventing aggregation and removing existing aggregates (281, 282). The use of successful immunization, passive and active, against pathological tau was first reported using an antibody targeting phospho-tau peptide encompassing the pS396/404 epitope. The passive and active use of this antibody therapy reduced pathological tau levels in mouse models, while attenuating behavioral phenotypes associated with tauopathies (283–285). In addition, the potential of an antibody-based therapy for tau pathology was investigated by Kfoury and colleagues who used a monoclonal antibody against the tau repeat domain, HJ9.3 (epitope 306–321), to inhibit the trans-cellular propagation of tau aggregation (181). Research has continued to investigate the use of antibody therapies to inhibit tau assembly and propagation, which has led to the inclusion of active and passive immunizations in clinical trials.

#### Active

Active immunization induces the host immune system to stimulate an antibody response and produces an immunological memory with a long-lasting effect (47). However, eliciting an immune response to a native protein poses the risk of adverse immune reactions, which may affect normal tau and its physiological functions (278). Active immunotherapy has been shown to be effective in reducing pathological tau assembly and the production of pathological tau aggregates (17–19, 283, 286, 287). AADvac1 is an active vaccine in clinical trials consisting of a synthetic peptide derived from amino acids tau 294–305 coupled to keyhole limpet hemocyanin with an N-terminal cysteine (19). It originated from *in vitro* studies of monoclonal antibody DC8E8, which binds to a 6 amino acid sequence motif (HXPGGG) present four times within the MTBR, preventing tau oligomerization (20). Transgenic tauopathy rat studies showed that immunization induced tau antibodies that bind to tau in the brain, reduced tau pathology and ameliorated behavioral deficits (19). Phase 1 trials of AADvac1 showed that 29 of 30 patients developed an immunoglobulin G response to the tau peptide component of AADvac1 and against pathological tau 151–391/4R. Two patients withdrew due to adverse events, one from a viral infection that lead to a seizure, thought to be due to the vaccine, and the other an episode of microhemorrhage in a patient with a history of this condition (21). The immunogenicity of AADvac1 has prompted a larger phase 2 trial, which is underway to further assess its safety and effectiveness in alleviating clinical symptoms, and its effect on AD biomarkers in the blood and CNS (278).

#### Passive

In passive immunization, monoclonal antibodies or immune serum are administered directly to subjects. This tends to offer a more transient effect than active immunization, but can be useful in minimizing risks associated with active immunization (278). The use of passive immunization has been shown not to be limited to targeting extracellular tau. Studies have shown that anti-tau antibodies are able to penetrate the blood brain barrier and also to enter neurons, where they can target intracellular pathological tau and tau aggregates (17, 22, 283, 288–290). Having seen promising results with passive immunization, a number of vaccines targeting various tau epitopes are being developed. BIIB092 (also known as BMS-986168) is a vaccine currently focused as a PSP treatment, rather than AD. It targets an N-terminal fragment present in extracellular tau (eTau) which was originally observed to be secreted from neurons derived from stem cells of familial AD patients (291). Bright and colleagues showed a correlation between eTau and Aβ *in vitro* and *in vivo*, whereby addition of eTau induced hyperactivity of neurons and increased Aβ production, whereas targeting eTau with an antibody recognizing residues 9–18 of eTau blocked these effects. Two phase 1 trials were completed in patients with PSP and healthy individuals and there is now a Phase 2 trial consisting of 400 patients with PSP. RG7345 is a humanized antibody that targets a specific tau phosphorylated epitope at S422 that has been found enter neurons and reduce tau pathology in transgenic mice (289). However, after a Phase 1 trial it was discontinued by its developer (Biogen), presumably due to an unfavorable pharmacokinetic profile, as it was showing a good safety profile (278).

## Discussion

The inability of recombinant full-length tau to self-assemble has limited its experimental use and has created a considerable challenge in tauopathy research. To overcome this problem, research groups have developed different *in vitro* tau models in the hope of replicating the pathological aggregation of tau observed in tauopathies. However, this has produced a number of inconsistent findings, such as the contribution of PTMs to assembly propensity and the importance of intrinsic tau features in the assembly process. This illustrates the problem of the lack of a general *in vitro* tau model tau self-assembly. The recent cryo-EM studies revealing the structures core of filaments associated with tauopathies have further highlighted the complexity of tau self-assembly and the challenge in developing a reliable *in vitro* tau model. Development of a generally agreed model that consistently produces a reliable representation of pathological tau aggregation will aid in identifying and understanding the key features and mechanisms involved in tau self-assembly. This is essential for identifying therapeutic targets for the development of effective disease-modifying treatments.

## Author Contributions

SO wrote the manuscript. YA-H, MBM, KEM, CRH, and CMW contributed to the manuscript text. KEM and LCS prepared the figures. LCS managed the work. All authors contributed to the article and approved the submitted version.

## Conflict of Interest

The authors declare that this study received funding for YA-H, KEM, and SO from TauRx Therapeutics Ltd. an affiliate of WisTa Laboratories Ltd. The funder had the following involvement in the study: authors CRH and CMW hold office at TauRx Therapeutics Ltd. an affiliate of WisTa Laboratories Ltd. These authors contributed to writing and editing of the review manuscript and the decision to submit for publication. The remaining authors declare that the research was conducted in the absence of any commercial or financial relationships that could be construed as a potential conflict of interest.

## Abbreviations

AD: Alzheimer's disease
4R: four-repeats
3R: three-repeats
FTDP-17: frontotemporal dementia with parkinsonism-linked to chromosome 17
Aβ: amyloid beta
FTD: frontotemporal dementia
PiD: Pick's disease
CTE: chronic traumatic encephalopathy
CBD: corticobasal degeneration
PART: primary age-related tauopathy
PSP: progressive supranuclear palsy
AGD: argyrophilic grain disease
cryo-EM: cryo-electron microscopy
NFT: neurofibrillary tangle
PHF: paired helical filament
SF: straight filament
mAb: monoclonal antibody
bvFTD: behavioral-variant frontotemporal dementia
NPF: narrow Pick filament
WPF: wide Pick filament
PTM: post-translational modification
MTBR: microtubule-binding region
TAI: tau aggregation inhibitor
MT: methylthioninium
LMT: leuco-methylthioninium
MTC: methylthioninium chloride
LMTM: leuco-methylthioninium bis(hydromethanesulfonate).
